# Some Improvements on Good Lattice Point Sets

**DOI:** 10.3390/e26110910

**Published:** 2024-10-27

**Authors:** Yu-Xuan Lin, Tian-Yu Yan, Kai-Tai Fang

**Affiliations:** 1 Research Center for Frontier Fundamental Studies, Zhejiang Lab, Kechuang Avenue, Zhongtai Sub-District, Yuhang District, Hangzhou 311121, China; yuxuanlin@zhejianglab.com; 2 Guangdong Provincial Key Laboratory of Interdisciplinary Research and Application for Data Science, BNU-HKBU United International College, Zhuhai 519087, China; 3 School of Mathematics, Renmin University of China, No.59, Zhongguancun Street, Haidian District, Beijing 100872, China; yantianyu@ruc.edu.cn; 4 Department of Statistics and Data Science, Faculty of Science and Technology, BNU-HKBU United International College, 2000 Jintong Road, Tangjiawan, Zhuhai 519087, China

**Keywords:** entropy, Frobenius distance, generalized good lattice point set, good lattice point set, Kullback–Leibler divergence, Kriging model, linear level permutation, max-min distance, mixture discrepancy, representative points, threshold accepting algorithm

## Abstract

Good lattice point (GLP) sets are a type of number-theoretic method widely utilized across various fields. Their space-filling property can be further improved, especially with large numbers of runs and factors. In this paper, Kullback-Leibler (KL) divergence is used to measure GLP sets. The generalized good lattice point (GGLP) sets obtained from linear-level permutations of GLP sets have demonstrated that the permutation does not reduce the criterion maximin distance. This paper confirms that linear-level permutation may lead to greater mixture discrepancy. Nevertheless, GGLP sets can still enhance the space-filling property of GLP sets under various criteria. For small-sized cases, the KL divergence from the uniform distribution of GGLP sets is lower than that of the initial GLP sets, and there is nearly no difference for large-sized points, indicating the similarity of their distributions. This paper incorporates a threshold-accepting algorithm in the construction of GGLP sets and adopts Frobenius distance as the space-filling criterion for large-sized cases. The initial GLP sets have been included in many monographs and are widely utilized. The corresponding GGLP sets are partially included in this paper and will be further calculated and posted online in the future. The performance of GGLP sets is evaluated in two applications: computer experiments and representative points, compared to the initial GLP sets. It shows that GGLP sets perform better in many cases.

## 1. Introduction

Number theory plays a significant role in information theory, providing fundamentals for many applications. Number-theoretic methods aim at deriving a point set uniformly scattered to replace the Monte Carlo (MC) method in the numerical evaluation of multiple integrals. Many methods have been proposed, including Korobov (1959) [1], Hua and Wang (1981) [2], Niederreiter (1992) [3], and Hlawka (1962) [4], to obtain a point set scattered as uniformly as possible over the unit hypercube $C^{s}={\left[0,1\right]}^{s}$, called number-theoretic net (NT-net). This net has been widely utilized in experimental designs, high-dimensional integration, statistical inference, and computer experiments. Computer experiments involving a large number of runs and/or factors have become increasingly prevalent in various fields, driven by rapid developments in computational techniques. A key goal of these experiments is to approximate the true underlying system with a simpler surrogate metamodel. An adequate design of experiments can significantly reduce the bias between the metamodel and the true model. Space-filling designs are the most popular type of designs for computer experiments. A space-filling design method aims to uniformly scatter the design points across the experimental domain, similar to the generation of NT-net. To measure the space-filling properties of a point set or design, many criteria are used, including the maximin distance (Jonhson et al., 1990 [5]) and mixture discrepancy (Zhou et al., 2013 [6]). The Kullback–Leibler (KL) divergence (Kullback and Leibler 1951 [7]) is suitable for measuring the divergence between the distribution of a point set and the uniform distribution. KL divergence is widely used in many applications of information theory, and it can be used to measure space-filling properties.

There are many estimation methods used for computing the KL divergence or relative entropy between two continuous multivariate distributions based on samples. The KL divergence between a distribution and a uniform distribution can be simplified to the problem of estimation of entropy. Let KL(f∥g) denote the KL divergence between two multivariate densities: f(x) and g(x). For an *s*-variate uniform distribution over a unit hypercube, denoted as $C^{s}={\left[0,1\right]}^{s}$, the *s*-variate density g(x)=1 for $x\in C^{s}$. Therefore,

$$KL\left(f∥g\right)=\int f\left(x\right)log\frac{f(x)}{g(x)}dx=\int f\left(x\right)log\left(f\left(x\right)\right)dx=-H\left(x\right),$$

where H(x) represents the entropy of the continuous random variable x. Joe (1989) [8] provided the estimator of functionals of a multivariate density, including the entropy function based on a kernel density estimate, such that an empirical distribution and numerical integration are not necessary. Hence, in our case, Joe’s estimator of the KL divergence between the distribution of a point set over a unit hypercube $X=\{x_{1},\ldots ,x_{n}\}$ and the uniform distribution is given by the following:

(1) $$\hat{KL}\left(f∥g\right)=\frac{1}{n}\sum_{i=1}^{n}log\left(\hat{f}\left(x_{i}\right)\right),$$

where $\hat{f}\left(x\right)$ is the kernel density estimate of f(x) with bandwidth h>0, and

$$\hat{f}\left(x\right)=\frac{1}{nh^{s}}\sum_{i=1}^{n}k\left(\frac{x-x_{i}}{h}\right),x\in R^{s},$$

in which *k* is an *s*-variate density. Joe (1989) [8] provided some suggested choices of bandwidths and kernels for s≤4. In this paper, we chose the normal kernel and Scott’s rule for bandwidth (Scott 2015 [9]), in which $h=n^{-\frac{1}{s+4}}$, and sometimes *h* is fixed with specifications in the tables of this paper.

The $L_{p}$-distance of an n×s design *D* is defined by $Min_{d_{p}}\left(D\right)=min\left\{d_{p}\left(x,y\right):x\neq y,x,y\in D\right\}$, p≥1, where $d_{p}\left(x,y\right)=\sum_{k=1}^{s}{\left|x_{k}-y_{k}\right|}^{p}$ as the $L_{p}$-distance of any two rows x and y in *D*. Remark that the definition of the $L_{p}$-distance between two rows of a design does not necessarily involve taking the $\frac{1}{p}$ power as in the Euclidean distance between two points and the design can be defined on $C^{s}$ or another finite field. The $L_{p}$-distance of a design, also known as the separation distance, is the minimal distance between pairs of design points. The goal of a maximin distance design is to maximize this separation distance. On the other hand, discrepancy, which measures uniformity, should be minimized to achieve a uniform design. Discrepancy has been widely used in number-theoretic methods to evaluate the uniformity of a set of points. Within the context of reproducing kernel Hilbert spaces, Hickernell (1998) [10] proposed generalized $L_{2}$-discrepancies including centered $L_{2}$-discrepancy (CD) and wrap-around $L_{2}$-discrepancy (WD). Zhou et al. (2013) [6] proposed mixture discrepancy (MD), and indicated that MD can overcome the shortcomings of CD and WD. For a point set in the domain of a unit hypercube $C^{s}={\left[0,1\right]}^{s}$ that can be expressed as a matrix $X={\left(x_{ik}\right)}_{n\times s}$, the square value of MD is defined by the following:

(2) $$\begin{array}{cc} \; & MD^{2}\left(X\right)=\frac{1}{n^{2}}\sum_{i=1}^{n}\sum_{j=1}^{n}\prod_{k=1}^{s}(\frac{15}{8}-\frac{1}{4}|x_{ik}-\frac{1}{2}|-\frac{1}{4}|x_{jk}-\frac{1}{2}|-\frac{3}{4}\left|x_{ik}-x_{jk}\right|+\\ \; & \frac{1}{2}|x_{ik}-x_{jk}{|}^{2})-\frac{2}{n}\sum_{i=1}^{n}\prod_{k=1}^{s}(\frac{5}{3}-\frac{1}{4}|x_{ik}-\frac{1}{2}|-\frac{1}{4}|x_{ik}-\frac{1}{2}{|}^{2})+{\left(\frac{19}{12}\right)}^{s}. \end{array}$$

To evaluate the discrepancy of a design $D=(d_{ik})$ in which the levels are labeled by integers, the elements can be linearly transformed to $X=\left(x_{ik}\right)\in C^{s}$ by $x_{ik}=\left(d_{ik}-0.5\right)/n$. However, the computational complexities of MD and $L_{p}$-distance result in significant time consumption when *n* or *s* is too large. Instead, to evaluate the uniformity of the point set X, this paper adopts the Frobenius distance (FD); see Horn and Johnson (2012) [11]. A natural uniform condition on X is that the mean vector is 0 with a covariance matrix close to $\frac{1}{12}I_{s}$, where $I_{s}$ is the identity matrix in $R^{s}$. The uniformity of X can be measured by the Frobenius distance between the two matrices, the covariance matrix of X denoted by $A=(a_{ik})$ and $B=\left(b_{ik}\right)=\frac{1}{12}I_{s}$, as follows:

(3) $$FD\left(X\right)={∥A-B∥}_{F}=\sqrt{trace{\left(A-B\right)}^{T}\left(A-B\right)}={\left[\sum_{i=1}^{n}\sum_{k=1}^{s}{\left(a_{ik}-b_{ik}\right)}^{2}\right]}^{1/2}.$$

Since the calculation of FD in this paper involves point sets with large *n*, it is not necessary to transform the design *D* into X for computational convenience.

The good lattice point (GLP) set proposed by Korobov (1959) [1] is the most popularly used method for generating an NT-net. Given a generating vector $h=(h_{1},\ldots ,h_{s})$ satisfying $1\leq h_{k}<n,\;h_{j}\neq h_{k}$ for j≠k, and the greatest common divisor $gcd(h_{k},\;n)=1$, so that each column is a permutation of {1,…,n}, the design $D=(d_{ik})$ with

$$d_{ik}=ih_{k}\left(mod\;n\right),\;i=1,\ldots ,n,\;k=1,\ldots ,s,$$

is called the lattice point set of the generating vector h, where the multiplication operation modulo *n* is modified such that the result falls within the range [1,n]. Without loss of generality, $h_{1}$ is always set to be 1. If *D* has the minimal discrepancy among all possible generating vectors, it is called a good lattice point set. Hua and Wang (1981) [2] indicated that GLP sets tend to have a lower discrepancy. Searching for a GLP in large *n* and *s* requires long calculations. Saltykov (1963) [12] provided tables of suggested generating vectors for GLP sets for 3≤s≤10 and 100<n≤ 155,093 according to Korobov’s method (Korobov 1959 [1]). Saltykov’s tables have been widely utilized in numerical analyses. Hua and Wang (1981) [2] further extended these tables to include more values of *n* and *s*. Fang and Wang (1994) [13] attached the extended tables as appendices, and further developed the applications of GLP sets in statistics, including experimental designs and representative points. However, the space-filling properties of GLP sets can be further improved so that the tables of suggested generating vectors of GLP sets can be updated.

To study the space-filling properties of GLP sets, Zhou and Xu (2015) [14] treated the GLP set as a special class of regular designs. Therefore, the theorem by Zhou and Xu (2015) [14] stating that a linear-level permutation of a regular design does not decrease the $L_{p}$-distance also applies to GLP sets. The linear-level permuting design is defined by the following:

(4) $$D_{u}=D+1_{n}u=\left\{x_{i}+u,x_{i}\in D\right\}\;\left(mod\;n\right),\;i=1,\;\ldots ,\;n,$$

with a given permutation vector $u=(u_{1},\;\ldots ,\;u_{s})$, and $u_{k}\in \left\{0,\;1,\;\ldots ,\;n-1\right\},\;k=1,\;\ldots ,\;s$. Qi et al. (2018) [15] defined the best design under a certain space-filling criterion among the linear level permuting designs of a GLP set as the generalized good lattice point (GGLP) set. They noted that a specific type of permuting vector $u^{\left(i\right)}=\left(i,\;\ldots ,\;i\right),\;i\in \left\{1,\;\ldots ,\;n-1\right\}$ often results in better $L_{p}$-distances. With a given acceptable number of searches, Qi et al. (2018) [15] considered n−1$u^{\left(i\right)}$ vectors of this special type and then conducted a random search for all possible u vectors, fixing the first element $u_{1}$ as 0. Searching for the best permuting vector is a combinatorial optimization problem with $n^{s-1}$ possibilities. When *n* and *s* are large, a simple random search is not efficient; instead, a stochastic optimization algorithm is adequate. The threshold accepting (TA) algorithm proposed by Dueck and Scheuer (1990) [16], as a variation of the simulated annealing algorithm, has solved many NP-hard optimization problems. It has been successfully applied in constructing uniform designs, as noted by Fang et al. (2000) [17]. There are many adjustments for TA settings, among which, we consider the version by Fang et al. (2017) [18].

To update the tables of generating vectors for GLP sets, this paper considers the best special permuting vector from n−1 searches as a benchmark and then searches for the optimal u in the form of $\left(0,\;u_{2},\;\ldots ,\;u_{s}\right)$ using the TA algorithm. In Section 2, we provide a brief review of GGLP, including related basic theoretical evidence according to Zhou and Xu (2015) [14] and Qi et al. (2018) [15]. The details of conducting the TA algorithm to search for GGLP sets are elaborated in Section 3. With the given initial GLP sets provided in work by Fang and Wang (1994) [13], the resulting GGLP sets, searched under the Frobenius distance, are evaluated by mixture discrepancy, maximin distance, and KL divergence in Section 4. According to the KL divergence, the distributions of GGLP sets and the initial GLP sets are very close, as the KL divergence is nearly the same when *n* is large. For small *n*, most GGLP sets exhibit lower KL divergence. The mixture discrepancy of GGLP sets can be improved for all *n*. Moreover, we also compare the performances in several case studies, including representative points and computer experiments. For a computer experiment, we generate training data according to pairs of GLP and GGLP sets from the Wood function, and predict a randomly generated testing set with the Kriging modeling technique. As for the application of representative points, we utilize the Box–Muller method to transform the points generated with Monte Carlo, GLP, and GGLP methods, respectively, into quasi-Monte Carlo points of bivariate mixture normal distribution, and use them in bootstrap resampling to approximate parameters of the distribution. The performances are evaluated by the mean squared error. In many cases, GGLP sets outperform GLP sets.

## 2. Review on Generalized Good Lattice Point Sets

Zhou and Xu (2015) [14] specified that a regular $q^{s-r}$ design *D* with *q* levels for each factor can be constructed by D=MG, where *M* is the $q^{s-r}$ full factorial design over the finite ring $Z_{q}=\left\{0,\;1,\;\ldots ,\;s-1\right\}$ and *G* is a generating matrix. A regular $q^{s-r}$ design defined in such a way has s−r independent columns spanning an (s−r)-dimensional linear space over the ring $Z_{q}$ and *r*-dependent columns specified in *G*. For any row, $x=(x_{1},\;\ldots ,\;x_{s})\in D$, the $L_{p}$-Lee weight is defined as $Lee_{p}\left(x\right)=\sum_{k=1}^{s}{\left\{min\left(x_{k},\;s-x_{k}\right)\right\}}^{p}$, implying that $Lee_{p}(|x-y|)\leq d_{p}\left(x,\;y\right)$, where $\left|x-y|=(|x_{1}-y_{1}|,\;\ldots ,\;|x_{s}-y_{s}|\right)$. Similar to the $L_{p}$-distance of *D*, the $L_{p}$-Lee weight of a design *D* is defined as $Min_{Lee_{p}}\left(D\right)=min\left\{Lee_{p}\left(x\right):\;x\neq 0,\;x\in D\right\}$. Zhou and Xu (2015) [14] have proved the following Lemmas 1 and 2.

**Lemma** **1.** *The $L_{p}$-distance of a regular $q^{s-r}$ design D over $Z_{q}$ is $Min_{d_{p}}\left(D\right)=Min_{Lee_{p}}\left(D\right)$.*

**Lemma** **2.** *For a regular $q^{s-r}$ design D over $Z_{q}$, a linear level permutation does not decrease the $L_{p}$-distance, i.e., $Min_{d_{p}}\left(D_{u}\right)\geq Min_{d_{p}}\left(D\right)$ where $D_{u}$ is as defined in* (4).

A GLP set can be considered a type of regular design featuring an independent column $M={\left(0,\;1,\;\ldots ,\;n-1\right)}^{T}$ and the generating matrix G=h. The only difference between GLP sets and regular designs is the replacement of 0 with *n* in GLP sets. Zhou and Xu (2015) [14] indicated that the special regular design, i.e.,

(5) $$D_{0}=Mh\;\left(mod\;n\right),$$

as well as the corresponding GLP set *D* derived by replacing the first row (0,…,0) of $D_{0}$ with row (n,…,n), have the same $L_{p}$-distance.

**Proposition** **1.** *The regular design*  $D_{0}$ *defined in* (5) *and the resulting good lattice point set D have the same $L_{p}$-distance, and according to Lemma 1,*

$$Min_{d_{p}}\left(D\right)=Min_{d_{p}}\left(D_{0}\right)=min\left\{Lee_{p}\left(x\right):\;x\neq n1_{s}^{T},\;x\in D\right\}.$$

Considering a GLP set as a special case of regular designs, Qi et al. (2018) [15] provided the following corollary:

**Corollary** **1.** *Given an n×s good lattice point set D, the linear level permutation of D does not decrease the $L_{2}$-distance, i.e., $d_{2}\left(D_{u}\right)\geq d_{2}\left(D\right)$, where $D_{u}$ is defined as* (4).

Zhou and Xu (2015) [14] also provided the upper bound of $L_{p}$-distance for any *p*. For a given initial GLP set, the linear level permuting design with the maximal $L_{2}$-distance or with the minimal MD is called the generalized good lattice point set (Qi et al., 2018 [15]). When exploring the best permuting vector u, it is sufficient to permute the s−1 dependent column while keeping the first independent column unchanged, allowing the first element of u to be fixed as 0. However, Qi et al., 2018 [15] found that the special permuting vector $u^{\left(i\right)}$ increases the $L_{p}$-distance for p=2 in most cases. Hence, when limiting the number of searches to *K* times with a given initial GLP set, they first considered the n−1 special $u^{\left(i\right)}$ vector and then conducted a random search for u of the form $u=(0,\;u_{2},\;\ldots ,\;u_{s})$. We incorporate the threshold-accepting algorithm for searching the best permuting vector by taking the best special $u^{\left(i\right)}$ as a benchmark. Since the tables of GLPs in Fang and Wang (1994) [13] refer to large *n* and *s*, the time consumption of the MD or $L_{2}$-distance is not admissible. Instead, when conducting the TA algorithm, this paper considers the Frobenius distance defined in (3). When *n* is large, the KL divergence of GGLP sets is almost the same as the initial GLP sets. This indicates that the distributions of GLP and GGLP sets are very close. Hence, the KL divergence defined in (1) is used only for comparing the results and is not considered a criterion of the TA algorithm.

## 3. Modified Threshold Accepting Algorithm

To find the best linear level permuting design of a given GLP set denoted as $X_{0}$, the threshold accepting algorithm starts with a random initial permuting vector, say $u_{0}$, as the current solution $u_{c}$, and then randomly chooses a neighborhood of $u_{c}$ as a candidate new solution $u_{n}$. The neighborhood of $u_{c}$ is defined by mutating one randomly chosen element $u_{k}^{c}$ from $u_{c}$, k=2,…,s, satisfying $u_{k}^{n}\neq u_{k}^{c}$ and $u_{k}^{n}\in \left\{0,\;1,\;\ldots ,\;n-1\right\}$. Here, *k* starts from 2 since the first element of u is fixed as 0 and remains unchanged during iterations. We denote the corresponding permuting designs of $X_{0}$ as $X_{u_{c}}$ and $X_{u_{n}}$, given by (4). Under the current threshold, $T_{i}\geq 0$, from a predetermined sequence, $T_{1}>\ldots >T_{I}=0$, the current solution is updated by $X_{n}$ if $f\left(X_{n}\right)-f\left(X_{c}\right)\leq T_{i}$ in the minimization problem or $f\left(X_{c}\right)-f\left(X_{n}\right)\leq T_{i}$ in the maximization problem, where f(·) represents any space-filling criterion. After *J* iterations of searches, the threshold is updated, and the procedure is repeated. The final threshold is set to 0, which implies a transition to a greedy algorithm. In this paper, we adopt the diminishing manner of threshold sequence and the historical optimum reversion proposed by Fang et al. (2017) [18]. Moreover, we include a uniqueness mechanism to ensure that each candidate new solution, represented by the linear-level permuting vector u, only appears once.

### 3.1. Threshold Sequence and Historical Optimum Reversion

In the literature, the threshold sequence of TA is always data-driven and diminishes exponentially, ending at 0. With a specified space-filling criterion f(·), *J* random linear level permuting vectors are generated, and the f(·) values of the *J* resulting permuting designs are measured. Let *R* denote the range of f(·) values. With a predetermined ratio 0<α<1, the first threshold is given by $T_{1}=\alpha R$. If the sequence diminishes in an exponential manner, the thresholds are defined as follows:

$$T_{i}=\frac{I-i}{I}T_{i-1},\;i=2,\;\ldots ,\;I,$$

where *I* is the number of thresholds. Fang et al. (2017) [18] found that the exponential diminishing manner approaches 0 too early so that the last several thresholds are all 0s. Therefore, Fang et al. (2017) [18] adopted a mixture diminishing manner in which the sequence first diminishes exponentially, and then starts to diminish linearly if $T_{i}\leq cT_{1}$, 0<c<1, given by

$$T_{i}=\frac{I-i-1}{I-i}T_{i-1}.$$

Moreover, in Fang et al. (2017) [18], the best solution is recorded and updated during the entire search process, and the current solution is replaced by the best solution before updating the threshold. This mechanism avoids the situation in which TA visits the best solution and then slips away in the following iterations.

### 3.2. Flowchart

**Figure 1:**
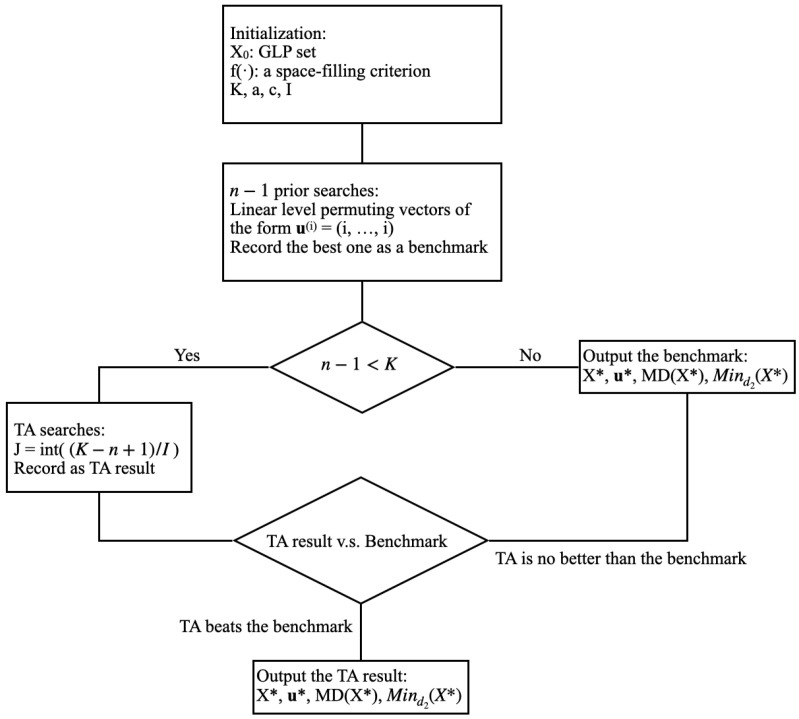
The flowchart of modified TA for GGLP.

With the initially specified GLP set $X_{0}$, a space-filling criterion f(·), threshold parameters α=0.1, c=0.03, I=10, and the number of total searches $K=10^{4},5\times 10^{4},10^{5}$ depending on the case, the flowchart of the modified TA algorithm for GGLP is presented in Figure 1.

The n−1 searches are first conducted for the special type of permuting vectors $u^{\left(i\right)}$. The best one among them under a specified space-filling criterion is recorded as a benchmark. With large *n* and *s*, the Frobenius distance in (3) is adopted as the criterion while the MD or maximin distance can be adopted for small-sized cases. The TA result is compared with the benchmark under the given criterion. The final GGLP set is also evaluated by the MD and $L_{p}$-distance for p=2. Since we adopt a uniqueness mechanism, the number of searches in the TA algorithm, I×J, should be less than $n^{s-1}$, total possibilities for permuting vectors. The comparisons of the resulting GGLP sets with the initial GLP sets are illustrated in the next section.

## 4. Comparisons Between GLP and GGLP Sets in Space-Filling Criteria and Applications

In this section, we compare the space-filling properties in terms of MD, maximin distance, and KL divergence between the initial GLP sets and the resulting GGLP sets obtained from our approach, with respect to $L_{2}$-distance $Min_{d_{2}}\left(\cdot \right)$, MD, and FD, respectively. We can see that MD can be improved by our algorithm with respect to MD or FD. In most cases of small-sized *n*, GGLP sets have lower KL divergence from the uniform distribution than the initial GLP sets. Frobenius distance is easy to calculate, especially for large *n*. The maximin distance $Min_{d_{2}}\left(\cdot \right)$ is only efficient in cases with small-sized *n*. To evaluate the performances of GGLP sets in practice, we utilize pairs of initial GLP sets and the resulting GGLP sets in two applications of computer experiments and representative points, respectively.

### 4.1. Comparisons in Maximin Distance, Mixture Discrepancy, and KL Divergence

The findings of this subsection are listed below.

1. The initial GLP sets are good enough and even better than the GGLP sets in the literature.
2. For small-sized *n*, our algorithm can improve both MD and maximin distance. When using $Min_{d_{2}}\left(\cdot \right)$ as the criterion of our algorithm, both criteria can be improved in most cases. However, when using MD as the criterion, $Min_{d_{2}}\left(\cdot \right)$ cannot be improved in most cases, except for large *s*.
3. Most of the resulting GGLP sets for small-sized *n* have lower KL divergence from the uniform distribution than the initial GLP sets. For large-sized *n*, the KL divergence of GGLP sets and the initial GLP sets is nearly the same.
4. When *n* is large, $Min_{d_{2}}\left(\cdot \right)$ is unchanged so that it is no longer suitable to be considered as a criterion.
5. For large *n*, using the Frobenius distance as the criterion can improve MD, which is not better than the MD when using MD as the criterion, but is better than the MD when using $Min_{d_{2}}\left(\cdot \right)$ as the criterion.
6. The Corollary 1 does not hold for MD, as we find a contradictory case where the MD of linear-level permuting design is worse than the initial GLP sets.
7. The number of searches *K* hardly affects the result of our algorithm, and even larger *K* sometimes leads to worse results.

For small-sized *n* and *s*, the criterion of the TA algorithm can be set as MD or the maximin distance with p=2, i.e., the $L_{2}$-distance $Min_{d_{2}}\left(\cdot \right)$. Table 1 shows the comparisons of $Min_{d_{2}}\left(\cdot \right)$, MD, and KL divergence among the initial GLP sets in the work by Fang and Wang (1994) [13], and the resulting GGLP sets with respect to $Min_{d_{2}}\left(\cdot \right)$ and MD, respectively.

The *K* in Table 1 is $10^{4}$, except for the 29×4 setting $K=1.5\times 10^{4}$. The best u of the form [i] in Table 1 represents that the best result comes from the special type of permuting vector $u^{\left(i\right)}$ while the best u starting from 0 is produced by the TA algorithm. For n=17 and s=8, Qi et al. (2018) [15] provided a GGLP set with $Min_{d_{2}}\left(\cdot \right)=204$ and MD=0.87 under either maximin distance or MD, using an initial GLP set with $Min_{d_{2}}\left(\cdot \right)=204$ and MD=0.89. In our case, the initial 17×8 GLP set is already better than the resulting GGLP set of Qi et al. (2018) [15]. Our algorithm can further improve the two space-filling criteria. The KL divergence of the resulting GGLP sets is also improved in most cases. With respect to MD, our algorithm cannot improve the $L_{p}$-distance most of the time but can provide better MD. If taking maximin distance as the criterion, our algorithm can simultaneously improve the $L_{p}$-distance and MD in most cases, but sometimes, MD may be worse as in the case of the 13×5 GGLP set. It also indicates that Corollary 1 does not hold for MD. Similarly, the KL divergence is improved, except for the 13×5 GGLP set. It indicates the consistency between mixture discrepancy and the KL divergence from the uniform distribution. More GGLP sets with respect to maximin distance and MD are included in Appendix A. For large *n*, the calculation of $L_{p}$-distance or MD is not admissible so we suggest adopting Frobenius distance in (3) instead. As for the KL divergence, the differences between GLP and GGLP sets are very small and can be considered computational errors. We first conduct our algorithm with respect to $Min_{d_{2}}\left(\cdot \right)$ and MD on the three illustrative cases, 1069×5, 3997×8, 4661×10 in Table 2, and then compare them with the results (with respect to the Frobenius distance) in Table 3.

In Table 2, the three cases are calculated with $K=10^{4}$ and $10^{5}$, respectively, and have the same results, except for 4661×10 with respect to MD. Among them, TA hardly beats the benchmarks. With 10 times larger K, the only TA result has improved slightly from 0.005262 to 0.005259. Hence, increasing *K* is not crucial in our case. The results of the three cases when using Frobenius distance (FD) as a criterion are listed in Table 3, where $Min_{d_{2}}\left(\cdot \right)$ and the MD of the initial GLP sets are the same as in Table 2, and will be omitted. $Min_{d_{2}}\left(\cdot \right)$ is hardly improved no matter the criterion. Even though the MD of GGLP sets under the Frobenius distance is no better than the one under MD, it is better than the MD of GGLP sets under maximin distance. Similarly, as shown in Table 2, taking a larger $K=10^{5}$ for the case 3997×8 even leads to a worse result. More GGLP sets with large *n* under Frobenius distance are included in Appendix A, and calculations will continue to be updated and posted online in the future.

### 4.2. Case Study

We conducted two applications to evaluate the performances of generalized good lattice point sets obtained in our approach, compared to the initial GLP sets. For a computer experiment, the number of experimental runs can be large since the experimental cost is often affordable. In the first case study, we use the Wood function as the true underlying model and adopt the Kriging modeling technique to obtain the metamodel. The performance of designs is evaluated by the mean squared error (MSE) of prediction. It turns out that the GGLP sets perform better in most cases. In the second case study, we combined the Box–Muller method with MC, GLP, and GGLP methods to generate QMC points for approximating a bivariate mixed normal distribution. The approximation performance was measured using the MSE of bootstrap resampling. The results indicate that GLP and GGLP points significantly outperform MC points, with GGLP often outperforming GLP.

#### 4.2.1. Computer Experiment of the Wood Function

The Wood function has been widely used to evaluate the performance of optimization algorithms, defined as follows:

$$\begin{array}{cc} y(x)= & 100{\left(x_{1}^{2}-x_{2}\right)}^{2}+{\left(1-x_{1}\right)}^{2}+90{\left(x_{4}-x_{3}^{2}\right)}^{2}+{\left(1-x_{3}\right)}^{2}\\ \; & +10.1\left({\left(x_{2}-1\right)}^{2}+{\left(x_{4}-1\right)}^{2}\right)+19.8\left(x_{2}-1\right)\left(x_{4}-1\right), \end{array}$$

where $x=\left(x_{1},\;x_{2},\;x_{3},\;x_{4}\right)\in {\left[-2,\;2\right]}^{4}$. Suppose there are *n* design points $x_{i},\;i=1,\;\ldots ,\;n$, and the Wood function gives outputs $y_{i}=y\left(x_{i}\right)$, the universal Kriging model is defined as follows:

$$y\left(x\right)=\sum_{j=0}^{L}\beta_{j}B_{j}\left(x\right)+z\left(x\right),$$

where the set of $B_{j}$ is a predetermined basis, and z(x) is a Gaussian process. The ordinary Kriging model is the most commonly used one with the form y(x)=μ+z(x), in which μ is the overall mean of $y_{i}$. We first obtain six pairs of 4-factor GLP and GGLP sets with different numbers of design points and their corresponding responses. The Kriging modeling is conducted using the *ooDACE* toolbox in MATLAB to obtain the metamodel, in which three kinds of bases are tried, respectively, i.e., the ordinary Kriging model denoted by Poly0, the polynomial basis with the first order, denoted by Poly1, and with the second order, denoted by Poly2. For each metamodel, the MSE between the predicted outputs of the same set of *M* random points and the true responses is calculated by the following:

$$MSE=\frac{1}{M}\sum_{m=1}^{M}{\left(y_{m}-{\hat{y}}_{m}\right)}^{2},$$

in which we take M=1000. The resulting MSE is listed in Table 4.

The prediction MSE does not simply decrease with a large number of runs, indicating the significance of the design in computer experiments. The observations are listed as follows:

1. The Kriging model with a second-order polynomial basis is more adequate for the Wood function.
2. The best result is produced by GGLP sets with n=3001 and the Kriging model with a second-order polynomial basis.
3. For n=562, with the Kriging models Poly0 and Poly1, GLP sets are better than GGLP sets. In all other cases, GGLP sets perform better.
4. The effectiveness of designs with n=701 and n=3001 is much better than others. The designs with n=701 are more effective than the ones with n=1019,2129.

#### 4.2.2. Quasi-Monte Carlo Points

Discrete approximations of continuous distributions are widely utilized, particularly when integration techniques fail to provide analytic solutions for complex multivariate distributions. Let x be a *p*-variate random vector following a continuous distribution F(x). The MC method generates a random sample $\left\{x_{1},\ldots ,x_{k}\right\}$ with an empirical distribution given by

$$F_{k}\left(x\right)=\frac{1}{k}\sum_{j=1}^{k}I\left\{x_{j}\leq x\right\},$$

where I{A} is the indicator function of event *A*. Under regular assumptions, the weak law of large numbers implies that $F_{k}\left(x\right)$ converges to F(x) in probability as k→∞. Therefore, $F_{k}\left(x\right)$ can be used to estimate F(x).

There are three popular methods for representative points, namely, the MC method, the QMC method, and the MSE method. The convergence rate for numerical integration by the MC method is $O\left(k^{-1/2}\right)$, independent of dimension *p*, which is slow. The mean squared error method provides a set of representative points by minimizing the MSE of the discrete approximation to a given continuous distribution. Another approach involves a set of points of size *k* generated by QMC methods, called quasi-random *F*-numbers (or QMC *F*-numbers) with respect to F(x) with a convergence rate of $O\left({\left(\log k\right)}^{p}/k\right)$, offering a faster alternative to the MC method when *p* is not large. Fang and Pan (2023) [19] provided evidence that representative points outperform random samples in approximating distributions. Simulation results indicate that QMC and MSE representative points perform better in most comparisons, suggesting that these representative points have significant potential in statistical inference.

Assume that the random vector $x∼F\left(x\right),x\in R^{p}$ has a stochastic representation:

$$x=h\left(y\right),\;y∼U\left(C^{s}\right),\;s⩽p,$$

where the random vector y is uniformly distributed over the unit cube $C^{s}$, and h is a continuous function on $C^{s}$. Utilizing number-theoretic methods to generate a uniformly scattered set of points $\left\{c_{i},i=1,\ldots ,n\right\}$ on $C^{s}$, let

$$x_{i}=h\left(c_{i}\right),\;i=1,\ldots ,n.$$

The points of $\left(x_{1},x_{2},\ldots ,x_{n}\right)$ are referred to as QMC points when F(x) is the uniform distribution on a *s*-dimensional unit cube $C^{s}$. We consider using MC, GLP, and GGLP points to generate QMC points to approximate multivariate mixed distributions. Let the cumulative distribution function (CDF) of *X* be denoted by $F_{x}$. Algorithms for generating quasi-random samples $\left(x_{1},x_{2},\ldots ,x_{n}\right)$ usually rely on a sequence of uniform quasi-random samples $\left(u_{1},u_{2},\ldots ,u_{n}\right)$. If the CDF $F_{x}$ is continuous, then given a uniform sample u, the desired sample x may be obtained by the formula

$$x=F_{x}^{-1}\left(u\right),$$

where $F_{x}^{-1}$ denotes the inverse function.

For the mixture of bivariate normal distributions, $MN_{2}\left(\alpha ,\mu_{1},\Sigma_{1},\mu_{2},\Sigma_{2}\right)$, the CDF $F_{x}\left(x\right)$ is given by the following:

(6) $$\begin{array}{c} F_{x}\left(x\right)=\alpha \cdot \phi \left(x|\mu_{1},\Sigma_{1}\right)+\left(1-\alpha \right)\cdot \phi \left(x|\mu_{2},\Sigma_{2}\right), \end{array}$$

where ϕ(x|μ,Σ) denotes the bivariate normal density function with mean vector μ and covariance matrix Σ.

If we use the same inverse transform method as before, namely, $x=F_{x}^{-1}\left(u\right)$, it is important to note that the CDF $F_{x}\left(x\right)$ of $MN_{2}\left(\alpha ,\mu_{1},\Sigma_{1},\mu_{2},\Sigma_{2}\right)$ has no simple closed form. Therefore, the inverse operation requires advanced numerical methods.

Box–Muller (1958) [20] proposed a transformation method that constructs samples from a normal distribution using samples uniformly distributed. If $\left(U_{1},U_{2}\right)$ are a pair of uniform and independent random variables in (0,1), then $\left(X_{1},X_{2}\right)$ are a pair of standard normal variables N(0,1), also independent, with $X_{1}$ and $X_{2}$ given by the following:

$$\begin{array}{cc} & X_{1}=\sqrt{-2\ln\left(U_{1}\right)}\cos\left(2\pi U_{2}\right),\\ & X_{2}=\sqrt{-2\ln\left(U_{1}\right)}\sin\left(2\pi U_{2}\right). \end{array}$$

To obtain a pair of variables $\left(Y_{1},Y_{2}\right)$ that follow a $N(\mu ,\sigma^{2})$ distribution, we use the following transformations:

$$\begin{array}{cc} & Y_{1}=\mu +\sigma X_{1},\\ & Y_{2}=\mu +\sigma X_{2}. \end{array}$$

Therefore, it is possible to generate QMC points from the $MN_{2}\left(\alpha ,\mu_{1},\Sigma_{1},\mu_{2},\Sigma_{2}\right)$ distribution using the Box–Muller method by generating uniformly distributed samples over a unit hypercube $C^{5}={\left[0,1\right]}^{5}$.

#### 4.2.3. The Algorithm to Generate QMC Points from $MN_{2}\left(\alpha ,\mu_{1},\Sigma_{1},\mu_{2},\Sigma_{2}\right)$

Based on the Box–Muller method, the problem of generating samples from the distribution $MN_{2}\left(\alpha ,\mu_{1},\Sigma_{1},\mu_{2},\Sigma_{2}\right)$ is transformed into the problem of sampling over a unit hypercube $C^{5}={\left[0,1\right]}^{5}$. We employ three different methods: MC, GLP, and GGLP methods to generate five-dimensional uniformly distributed random points over this unit hypercube. Consider the following algorithm to generate a random sample from $MN_{2}\left(\alpha ,\mu_{1},\Sigma_{1},\mu_{2},\Sigma_{2}\right)$.

**Step** **1.**  Find the positive definite square root $A_{i}$ such that $A_{i}^{T}A_{i}=\Sigma_{i},i=1,2$.
**Step** **2.**  Generate random numbers $u=(u_{1},u_{2},u_{3},u_{4},u_{5})$ uniformly distributed over a five-dimensional unit hypercube using MC, GLP, and GGLP methods.
**Step** **3.**  Use the Box–Muller method to calculate the corresponding standard normal variates $n_{1},n_{2},n_{3},n_{4}$.

$$\begin{array}{cc} r_{1}=\sqrt{-2\log\left(u_{1}\right)}, & r_{2}=\sqrt{-2\log\left(u_{3}\right)},\\ n_{1}=r_{1}\cos\left(2\pi u_{2}\right), & n_{2}=r_{1}\sin\left(2\pi u_{2}\right),\\ n_{3}=r_{2}\cos\left(2\pi u_{4}\right), & n_{4}=r_{2}\sin\left(2\pi u_{4}\right). \end{array}$$

**Step** **4.**  Calculate

$$N_{1}=\mu_{1}+A_{1}\left[\begin{array}{c} n_{1}\\ n_{2} \end{array}\right],\;\mathrm{and}\;N_{2}=\mu_{2}+A_{2}\left[\begin{array}{c} n_{3}\\ n_{4} \end{array}\right].$$

**Step** **5.**  Generate a two-dimensional random vector X. If $u_{5}\leq \alpha$, take $X=N_{1}$; otherwise, take $X=N_{2}$. Deliver X, which follows $MN_{2}\left(\alpha ,\mu_{1},\Sigma_{1},\mu_{2},\Sigma_{2}\right)$.
**Step** **6.**  Repeat steps 2 to 5 *n* times to obtain a set of QMC points $X_{1},\ldots ,X_{n}$ of $MN_{2}(\alpha ,\mu_{1},$$\Sigma_{1},\mu_{2},\Sigma_{2})$.

For a univariate mixture of normal distributions, denoted as $MixN(\alpha ,\mu_{1},\sigma_{1}^{2},\mu_{2},\sigma_{2}^{2})$, a MixN is a convex combination of two independent normal distributions with five parameters. The normal distribution belongs to the location-scale family. However, the convex combination of two normal distributions is no longer considered a location-scale distribution. There exist two notable subclasses that arise when the two normal components either share the same location parameter or possess identical scale parameters. Similarly, we can classify types of the bivariate mixture $MN_{2}\left(\alpha ,\mu_{1},\Sigma_{1},\mu_{2},\Sigma_{2}\right)$ based on these attributes.

- Scale mixture: $\mu_{1}=\mu_{2}=\mu$ and $\Sigma_{1}\neq \Sigma_{2}$;
- Location mixture: $\mu_{1}\neq \mu_{2}$ and $\Sigma_{1}=\Sigma_{2}=\Sigma$, where
  $$\Sigma =\left[\begin{array}{cc} \sigma_{11} & \sigma_{12}\\ \sigma_{21} & \sigma_{22} \end{array}\right]=\left[\begin{array}{cc} \sigma_{1}^{2} & \sigma_{1}\sigma_{2}\rho \\ \sigma_{1}\sigma_{2}\rho & \sigma_{2}^{2} \end{array}\right];$$
- Special case: A special case is when both mixture components have symmetric means and identical covariance matrices, where $\mu_{1}=-\mu_{2}=\mu$ and $\Sigma_{1}=\Sigma_{2}=\Sigma$;
- More specific case: A more specific case of the location mixture occurs when the correlation matrix is $R_{\rho }$, where $\sigma_{1}=\sigma_{2}=1$ and −1<ρ<1. $\mu_{1}=-\mu_{2}=\mu$ and $\Sigma_{1}=\Sigma_{2}=R_{\rho }$;

The parameter settings of distributions are given in Table 5.

The Bootstrap method, proposed by Efron (1994) [21], is a resampling technique widely used in statistical inference. Based on the four underlying distributions listed in Table 5, we use the Bootstrap method to evaluate the sampling effectiveness for three different methods and various types of $MN_{2}\left(\alpha ,\mu_{1},\Sigma_{1},\mu_{2},\Sigma_{2}\right)$. Given $X=\left\{x_{1},\ldots ,x_{n}\right\}$, which represents a set of *n* two-dimensional QMC points drawn from $MN_{2}\left(\alpha ,\mu_{1},\Sigma_{1},\mu_{2},\Sigma_{2}\right)$, the Bootstrap method is used to estimate the mean vector μ, and the covariance matrix Σ involves the following steps:

**Step** **1.**  Initialize i=1. Generate a sample $x_{i}^{*}=\left\{x_{1i},\ldots ,x_{ni}\right\}$ from $X=\left\{x_{1},\ldots ,x_{n}\right\}$.
**Step** **2.**  Calculate:

$${\hat{\mu }}_{i}=\frac{1}{n}\sum_{j=1}^{n}x_{ji}^{*},\;{\hat{\Sigma }}_{i}=\frac{1}{n}\sum_{j=1}^{n}\left(x_{ji}^{*}-{\hat{\mu }}_{i}\right){\left(x_{ji}^{*}-{\hat{\mu }}_{i}\right)}^{⊤}.$$

**Step** **3.**  Repeat Steps 1 and 2 for *B* iterations. Compute the estimated mean vector and covariance matrix of x as follows:

$$\hat{\mu }=\frac{1}{B}\sum_{i=1}^{B}{\hat{\mu }}_{i},\;\hat{\Sigma }=\frac{1}{B}\sum_{i=1}^{B}{\hat{\Sigma }}_{i}.$$

**Step** **4.**  Define the true population mean and covariance matrix of $MN_{2}\left(\alpha ,\mu_{1},\Sigma_{1},\mu_{2},\Sigma_{2}\right)$ as follows:

$$\mu =\alpha \mu_{1}+\left(1-\alpha \right)\mu_{2},\;\Sigma =\alpha \Sigma_{1}+\left(1-\alpha \right)\Sigma_{2}.$$

Evaluate the sampling effectiveness by calculating the MSE for the estimates:

$$MSE_{\mu }=\frac{1}{B}\sum_{i=1}^{B}{\left({\hat{\mu }}_{i}-\mu \right)}^{2},\;MSE_{\Sigma }=\frac{1}{B}\sum_{i=1}^{B}{\left({\hat{\Sigma }}_{i}-\Sigma \right)}^{2}.$$

In the following experiments, let B=5000. Use the calculated five-dimensional GLP and GGLP points to generate random samples of the corresponding sample size *n*, and the results are shown in Table 6 and Table 7.

As we can see in Table 6 and Table 7, when approximating the target distribution $MN_{2}\left(\alpha ,\mu_{1},\Sigma_{1},\mu_{2},\Sigma_{2}\right)$ using representative points generated by the MC, GLP, and GGLP methods, the GLP and GGLP methods show significant improvement in almost all cases compared to the MC method, particularly when the sample size *n* is small. In many instances, the GGLP method outperforms both the GLP and MC methods, particularly when the mixture type is a scale mixture. As *n* increases, the performance of the GGLP method is very similar to that of the GLP method, and both consistently outperform the MC method. Therefore, the sampling method based on the GGLP and Box–Muller techniques for $MN_{2}\left(\alpha ,\mu_{1},\Sigma_{1},\mu_{2},\Sigma_{2}\right)$ can more efficiently generate samples that approximate the target distribution.

## 5. Discussion

Under the maximin distance measurement, linear level permutation does not decrease the $L_{p}$-distance of GLP sets. Although this does not hold for mixture discrepancy, as we find some contradictory instances, GGLP sets can still improve the MD of GLP sets. According to the KL divergence from the uniform distribution, GGLP sets perform better than GLP sets in most small-sized cases and are nearly the same in large-sized cases. If maximin distance is used as a criterion, GGLP sets can simultaneously improve $L_{p}$-distance and MD for small-sized *n*. This indicates that the two criteria are consistent. In most large-sized cases, the maximin distance of initial GLP sets is hard to improve, while MD can be improved in all cases. However, for large-sized cases, calculating MD or $L_{p}$-distance is time-consuming. Instead, we adopt the Frobenius distance, which is easier to calculate, and the resulting GGLP sets also have lower MDs. Increasing the number of searches for large-sized GGLP sets is not necessary to improve results. The complete results with respect to the aforementioned three criteria are included in Appendix A. More results will be calculated and posted online in the future. To verify the performances of the resulting GGLP sets, we utilize the initial GLP sets and the corresponding GGLP sets in two applications of computer experiments and representative points. The advantage of GGLP sets in computer experiments is more significant. The initial GLP sets still maintain their effectiveness in the application of representative points, although in some cases, GGLP sets perform better.

## Figures and Tables

**Table 1:** GGLP sets with respect to $Min_{d_{2}}\left(\cdot \right)$ and MD, respectively.

*n*	*s*	Initial GLP Sets			GGLP Sets w.r.t. ${\mathrm{Min}}_{d_{2}}$				GGLP Sets w.r.t. MD			
		${Min}_{d_{2}}$	MD	KL	${Min}_{d_{2}}$	MD	KL	Best u	${Min}_{d_{2}}$	MD	KL	Best u
29	4	147	0.01	0.13 (0.6)	147	0.01	0.09 (0.6)	[28]	147	0.01	0.07 (0.6)	[0 1 1 21]
13	5	55	0.09	0.49	66	0.10	0.79	[0 6 11 9 11]	55	0.09	0.74	[0 7 9 8 7]
29	5	254	0.04	0.38	254	0.04	0.22	[28]	254	0.04	0.31	[0 17 26 3 27]
17	8	204	0.81	3.11	204	0.76	1.81	[16]	204	0.76	2.13	[0 1 12 1 5 12 2 5]
31	8	437	0.43	2.40	464	0.41	1.68	[0 3 25 26 9 13 18 14]	437	0.39	1.43	[7]
25	9	381	1.15	3.34	444	1.09	2.21	[0 14 5 17 5 23 5 20 12]	381	1.06	2.20	[0 17 21 11 9 7 4 14 18]
27	10	550	2.36	4.38	577	2.20	2.74	[0 23 14 8 19 24 9 11 10 25]	550	2.13	2.82	[0 9 21 17 24 21 22 9 21 24]
29	15	1016	58.34	7.65	1144	55.42	5.15	[0 13 16 10 22 9 12 21 18 28 12 4 8 14 7]	1045	54.87	4.57	[23]
31	15	1240	52.05	7.44	1426	49.59	4.50	[22]	1333	49.21	4.31	[7]

For KL divergence, the values in the parentheses indicate the fixed bandwidth, and the bandwidth adopts Scott’s rule if no parenthesis.

**Table 2:** Three illustrative GGLP sets with respect to $Min_{d_{2}}\left(\cdot \right)$ and MD, respectively.

*K*	*n*	*s*	Initial GLP Sets			GGLP Sets w.r.t. $d_{2}$			GGLP Sets w.r.t. MD		
			${Min}_{d_{2}}$	MD	KL	${Min}_{d_{2}}$	MD	Best u	${Min}_{d_{2}}$	MD	Best u
$10^{4}$	1069	5	41,010	0.0002368	0.81 (0.3)	41,010	0.0002196	[1068]	41,010	0.0001757	[549]
	3997	8	1,951,944	0.001573	0.10	1,951,944	0.001558	[3996]	1,951,944	0.001278	[3085]
	4661	10	3,565,998	0.006846	0.92	3,565,998	0.006722	[4660]	3,565,998	0.005262	[0 4341 3652 1461 1551 3665 3348 3082 167 3343]
$10^{5}$	4661	10							3,565,998	0.005259	[0 3781 3492 598 4249 3434 3656 1060 2896 2627]

The blank cells are the same as the ones above. The KL divergence of the GGLP sets is almost the same as the GLP sets, so it will be omitted. For KL divergence, the values in parentheses indicate a fixed bandwidth, and the bandwidth adopts Scott’s rule if there are no parentheses.

**Table 3:** Three illustrative GGLP sets with respect to the Frobenius distance.

*K*	*n*	*s*	Initial GLP Sets	GGLP Sets w.r.t. Frobenius Distance				
			FD	${Min}_{d_{2}}\left(\cdot \right)$	MD	FD	KL	**Best u**
$10^{5}$	1069	5	213,516.31	41,010	0.0001767	213,140.01	0.81 (0.3)	[557]
$5\times 10^{4}$	3997	8	3,771,719.97	1,951,944	0.001280	3,766,576.88	0.10	[0 2896 272 2747 2776 2399 3598 516]
$10^{5}$	3997	8		1,951,944	0.001282	3,766,577.57	0.10	[0 2888 3448 685 555 2157 560 3164]
	4661	10	5,729,496.43	3,565,998	0.005328	5,726,228.42	0.91	[0 4647 3322 1819 3264 2552 3289 4447 3926 2730]

For KL divergence, the values in the parentheses indicate the fixed bandwidth and the bandwidth adopts Scott’s rule if no parenthesis.

**Table 4:** The prediction MSE of Kriging models with different designs.

n	307		562		701		1019		2129		3001	
Design	GLP1	GGLP1	GLP2	GGLP2	GLP3	GGLP3	GLP4	GGLP4	GLP5	GGLP5	GLP6	GGLP6
Poly0	242.80	139.74	134.22	166.04	34.88	20.96	236.20	138.00	125.59	97.24	37.69	19.75
Poly1	230.72	125.03	121.40	232.32	35.74	20.33	214.75	124.83	115.06	88.67	33.78	17.68
Poly2	72.60	41.96	38.11	29.78	9.04	5.43	73.02	42.10	37.52	32.25	8.84	5.15

**Table 5:** Parameters of underlying distributions for the simulation.

Mixture Type	α	$\mu_{1}$	$\Sigma_{1}$	$\mu_{2}$	$\Sigma_{2}$
Scale	0.7	${\left[0,1\right]}^{T}$	[1,0.5;0.5,2]	${\left[0,1\right]}^{T}$	[1,−0.3;−0.3,1.2]
Location	0.7	${\left[0,1\right]}^{T}$	[1,0.5;0.5,1]	${\left[3,4\right]}^{T}$	[1,0.5;0.5,1]
Special Case	0.7	${\left[-1,1\right]}^{T}$	[2,0.5;0.5,1]	${\left[1,-1\right]}^{T}$	[2,0.5;0.5,1]
More Special	0.7	${\left[-1,1\right]}^{T}$	[1,0.5;0.5,1]	${\left[1,-1\right]}^{T}$	[1,0.5;0.5,1]

**Table 6:** The MSE error of sampling results for $MN_{2}\left(\alpha ,\mu_{1},\Sigma_{1},\mu_{2},\Sigma_{2}\right)$ of different methods.

Mixture Type	n	μ Error			Σ Error		
		GLP	GGLP	MC	GLP	GGLP	MC
Scale Mixture	11	**0.12748**	0.16169	0.23356	**0.17351**	0.25061	0.43120
	13	**0.10704**	0.12190	0.20275	0.34470	**0.32199**	0.38910
	15	0.15576	**0.14204**	0.17667	**0.17465**	0.38802	0.34706
	17	**0.07377**	0.09142	0.15799	0.17487	**0.16459**	0.31083
	19	**0.09793**	0.10482	0.13581	**0.13254**	0.14704	0.28491
	21	0.08822	**0.07907**	0.12874	**0.12851**	0.15544	0.26249
	23	0.07562	**0.06526**	0.12206	0.15974	**0.12388**	0.25402
	25	0.07731	**0.07614**	0.10707	0.14548	**0.12469**	0.22479
	27	0.08461	**0.05706**	0.10016	**0.11576**	0.16483	0.20820
	29	0.07287	**0.06288**	0.08904	0.41562	**0.18520**	0.19639
	31	0.08679	0.08824	**0.08652**	0.16582	**0.14292**	0.18767
	1069	0.00139	**0.00133**	0.00252	0.00331	**0.00300**	0.00605
	1543	0.00103	**0.00092**	0.00176	0.00290	**0.00222**	0.00411
	2129	**0.00064**	0.00065	0.00131	**0.00151**	0.00156	0.00304
	3001	0.00047	**0.00047**	0.00092	0.00118	**0.00110**	0.00218
	4001	0.00035	**0.00035**	0.00069	0.00079	**0.00079**	0.00162
	5003	0.00028	**0.00028**	0.00055	0.00066	**0.00065**	0.00128
	6007	0.00023	**0.00023**	0.00045	**0.00054**	0.00055	0.00108
	8191	0.00017	**0.00017**	0.00034	0.00039	**0.00039**	0.00079
Location Mixture	11	**0.25931**	0.33428	0.52269	**1.09738**	2.68476	3.52069
	13	**0.20750**	0.21403	0.42412	2.12740	**2.06051**	3.52895
	15	0.32909	**0.29343**	0.36442	**2.84073**	5.41317	3.76739
	17	**0.16624**	0.17735	0.33933	**2.53576**	3.83108	3.57281
	19	**0.15407**	0.20759	0.29507	**2.93498**	4.26999	3.54642
	21	**0.14548**	0.17202	0.26786	**3.35914**	5.31888	3.67118
	23	0.13306	**0.12150**	0.24839	3.45216	**2.32513**	3.61538
	25	**0.10698**	0.13039	0.23017	**2.85425**	3.42801	3.63253
	27	0.10599	**0.10459**	0.20823	**2.34247**	2.77279	3.66441
	29	0.11341	**0.10896**	0.19780	3.14358	**2.55764**	3.65183
	31	**0.09836**	0.13888	0.18390	**2.99949**	4.55371	3.63388
	1069	**0.00273**	0.00281	0.00536	3.64580	3.57866	**3.57075**
	1543	**0.00193**	0.00197	0.00370	3.57872	**3.56773**	3.57322
	2129	0.00135	**0.00135**	0.00270	**3.55498**	3.59095	3.58118
	3001	0.00097	**0.00092**	0.00191	3.58023	3.60328	**3.56851**
	4001	**0.00072**	0.00073	0.00144	3.56631	**3.55425**	3.57361
	5003	0.00058	**0.00058**	0.00115	**3.53904**	3.57851	3.57453
	6007	**0.00049**	0.00051	0.00097	**3.56414**	3.57053	3.58156
	8191	**0.00035**	0.00036	0.00071	**3.56046**	3.57118	3.57603

The best performances with each method are highlighted in blue and bold.

**Table 7:** The MSE errors of sampling results for $MN_{2}\left(\alpha ,\mu_{1},\Sigma_{1},\mu_{2},\Sigma_{2}\right)$ of different methods.

Mixture Type	n	μ Error			Σ Error		
		GLP	GGLP	MC	GLP	GGLP	MC
Special Case	11	**0.25165**	0.29929	0.41374	**0.93131**	1.03096	1.43671
	13	**0.14974**	0.16602	0.34652	**0.58370**	1.08717	1.36398
	15	0.30921	**0.17577**	0.30313	1.00388	**0.77147**	1.23658
	17	0.14990	**0.14487**	0.26970	0.90135	**0.60154**	1.20171
	19	**0.13719**	0.18429	0.24269	**0.57281**	0.63472	1.14612
	21	**0.14613**	0.15287	0.21579	**0.86959**	1.77426	1.12139
	23	**0.10489**	0.11986	0.19608	**0.44374**	0.71008	1.08158
	25	0.10518	**0.09005**	0.18204	**0.49432**	0.78170	1.06981
	27	**0.09236**	0.09986	0.17313	**0.66306**	1.08487	1.06859
	29	0.10570	**0.09277**	0.15624	1.29616	**0.98171**	1.03050
	31	**0.10099**	0.16849	0.14794	0.85060	**0.83034**	1.03772
	1069	**0.00219**	0.00235	0.00441	**0.70415**	0.71414	0.71624
	1543	0.00161	**0.00161**	0.00308	**0.68028**	0.70536	0.71176
	2129	0.00112	**0.00112**	0.00217	**0.70624**	0.72143	0.71126
	3001	0.00079	**0.00078**	0.00160	**0.70609**	0.71331	0.70712
	4001	0.00061	**0.00060**	0.00116	0.70776	0.70845	**0.70659**
	5003	0.00047	**0.00047**	0.00093	**0.69904**	0.71285	0.70713
	6007	0.00040	**0.00040**	0.00077	0.70567	**0.70252**	0.70727
	8191	0.00029	**0.00029**	0.00058	**0.70397**	0.70424	0.70638
More Specific Case	11	**0.19322**	0.22715	0.31140	0.88204	**0.87351**	1.02618
	13	**0.12060**	0.12801	0.26512	**0.50493**	0.83128	0.98595
	15	0.21495	**0.12976**	0.24514	0.89707	**0.58971**	0.97239
	17	0.11166	**0.10329**	0.21124	0.83907	**0.53303**	0.92968
	19	**0.11837**	0.12825	0.18746	0.61687	**0.61167**	0.90584
	21	0.11345	**0.10998**	0.17328	**0.80174**	1.13989	0.91073
	23	**0.08771**	0.09285	0.15596	**0.50079**	0.80591	0.88328
	25	0.08587	**0.07018**	0.14421	**0.55242**	0.72346	0.88145
	27	**0.07520**	0.07940	0.13809	**0.62866**	1.11045	0.86447
	29	0.07548	**0.07133**	0.12559	**0.82362**	0.85441	0.84072
	31	**0.07811**	0.10886	0.11813	0.77192	**0.65358**	0.84658
	1069	**0.00173**	0.00179	0.00344	**0.70237**	0.71045	0.71045
	1543	0.00124	**0.00120**	0.00238	**0.69404**	0.70760	0.70679
	2129	0.00086	**0.00086**	0.00172	**0.70650**	0.71258	0.70838
	3001	0.00061	**0.00061**	0.00124	**0.70470**	0.70881	0.70590
	4001	0.00046	**0.00045**	0.00094	0.70701	**0.70673**	0.70691
	5003	0.00038	**0.00038**	0.00073	0.70276	**0.71045**	0.70600
	6007	0.00031	**0.00031**	0.00060	0.70517	**0.70328**	0.70642
	8191	0.00023	**0.00023**	0.00045	**0.70485**	0.70529	0.70610

The best performances with each method are highlighted in blue and bold.

## Data Availability

The original contributions presented in the study are included in this article; further inquiries can be directed to the corresponding authors. The computer codes of this article are uploaded online in a GitHub repository, GGLP project https://github.com/YuxuanLin8/Improved-Generalized-Good-Lattice-Point-Sets (accessed on 1 October 2024).

## Abbreviations

The following abbreviations are used in this manuscript:

MC	Monte Carlo
QMC	quasi-Monte Carlo
CD	centered $L_{2}$-discrepancy
WD	wrap-around $L_{2}$-discrepancy
MD	mixture discrepancy
GLP	good lattice point
GGLP	generalized good lattice point
TA	threshold accepting
FD	Frobenius distance
MSE	mean squared error
CDF	cumulative distribution function

## Appendix A

**Table A1:** GGLP sets with respect to $d_{2}\left(\cdot \right)$ and MD, respectively.

*K*	*n*	*s*	Initial GLP Sets		GGLP Sets w.r.t. $d_{2}\left(\cdot \right)$			GGLP Sets w.r.t. MD		
			$d_{2}\left(\cdot \right)$	MD	$d_{2}\left(\cdot \right)$	MD	Best u	$d_{2}\left(\cdot \right)$	MD	Best u
15,000	29	4	147	0.0111	147	0.0106	[28]	147	0.0101	[0 1 1 21]
	31	4	150	0.0105	150	0.0107	[30]	150	0.0099	[0 12 19 0]
$10^{4}$	11	5	55	0.1216	66	0.1190	[10]	55	0.1168	[7]
	13	5	55	0.0943	66	0.0971	[0 6 11 9 11]	55	0.0923	[0 7 9 8 7]
	15	5	71	0.1077	74	0.1048	[0 9 0 8 6]	71	0.0990	[0 14 7 14 2]
	17	5	75	0.0739	75	0.0668	[16]	75	0.0659	[0 12 13 2 12]
	19	5	99	0.0606	99	0.0587	[18]	99	0.0583	[0 13 18 8 4]
	21	5	110	0.0570	110	0.0540	[20]	110	0.0532	[0 5 7 13 14]
	23	5	134	0.0557	134	0.0521	[22]	134	0.0513	[17]
	25	5	125	0.0320	125	0.0312	[24]	125	0.0306	[20]
	27	5	206	0.0507	206	0.0446	[26]	206	0.0423	[0 1 21 25 8]
	29	5	254	0.0441	254	0.0420	[28]	254	0.0404	[0 17 26 3 27]
50,000	31	5	310	0.0555	310	0.0478	[30]	310	0.0446	[8]
$10^{4}$	1069	5	41,010	0.000237	41,010	0.000220	[1068]	41,010	0.000176	[549]
$10^{4}$	17	8	204	0.8135	204	0.7626	[16]	204	0.7616	[0 1 12 1 5 12 2 5]
	19	8	204	0.7183	240	0.6809	[0 15 13 6 12 1 9 9]	204	0.6642	[18]
	23	8	300	0.6088	335	0.5622	[0 6 13 22 15 0 8 13]	300	0.5409	[17]
	25	8	260	0.5480	365	0.5119	[0 23 1 11 19 13 24 0]	260	0.5005	[0 0 22 7 10 17 1 16]
	27	8	380	0.5002	428	0.4602	[0 9 2 1 21 11 14 1]	380	0.4468	[22]
	29	8	443	0.4960	467	0.4525	[0 17 24 18 8 2 4 26]	443	0.4346	[23]
	31	8	437	0.4328	464	0.4096	[0 3 25 26 9 13 18 14]	437	0.3942	[7]
	3997	8	1,951,944	0.001573	1,951,944	0.001558	[3996]	1,951,944	0.001278	[3085]
	17	9	205	1.8237	229	1.7667	[0 6 8 3 14 15 1 6 13]	205	1.7051	[0 2 12 9 5 8 2 12 12]
	19	9	285	1.5020	285	1.4023	[18]	285	1.4023	[18]
	23	9	349	1.2510	393	1.1682	[0 22 8 8 6 5 16 19 14]	349	1.1264	[7]
	25	9	381	1.1463	444	1.0929	[0 14 5 17 5 23 5 20 12]	381	1.0622	[0 17 21 11 9 7 4 14 18]
	27	9	549	1.0109	567	0.9546	[26]	549	0.9479	[0 26 9 23 2 7 1 9 9]
	29	9	468	1.0137	555	0.9484	[0 18 5 2 3 8 13 24 1]	468	0.9030	[23]
	31	9	548	0.8939	581	0.8395	[0 26 23 8 15 15 2 8 5]	548	0.8192	[25]
	19	10	286	3.2735	313	3.1116	[0 9 17 3 12 11 0 15 3 13]	289	3.0548	[2]
	23	10	385	2.5276	448	2.3573	[0 12 20 7 19 15 7 3 15 1]	385	2.2997	[17]
	25	10	525	2.3441	525	2.2001	[24]	525	2.1927	[0 9 18 7 21 17 3 23 21 20]
	27	10	550	2.3572	577	2.2038	[0 23 14 8 19 24 9 11 10 25]	550	2.1252	[0 9 21 17 24 21 22 9 21 24]
	29	10	568	2.0379	664	1.8879	[0 5 8 0 19 23 8 25 13 4]	568	1.8399	[23]
	31	10	597	1.8133	669	1.7315	[0 20 16 10 9 10 20 0 27 13]	597	1.6732	[25]
	4661	10	3,565,998	0.006846	3,565,998	0.006722	[4660]	3,565,998	0.005262	[0 4341 3652 1461 1551 3665 3348 3082 167 3343]
	29	15	1016	58.3406	1144	55.4192	[0 13 16 10 22 9 12 21 18 28 12 4 8 14 7]]	1045	54.8724	[23]
	31	15	1240	52.0468	1426	49.5877	[22]	1333	49.2098	[7]

The blank cells are the same as the above ones.

**Table A2:** GGLP sets with respect to the Frobenius distance.

*K*	*n*	*s*	Initial GLP Sets			GGLP Sets w.r.t. Frobenius Distance		
			$d_{2}\left(\cdot \right)$	MD	FD	MD	FD	Best u
15,000	29	4	147	0.0110525	153.38	0.0100892	144.92	[0 5 4 24]
	31	4	150	0.0104753	169.98	0.0099667	165.34	[0 20 14 10]
50,000	307	4	4245	0.0003195	15,789.38	0.0002848	15,759.36	[232 232 232 232]
	562	4	16,860	0.0000640	52,744.84	0.0000610	52,734.18	[551 551 551 551]
	701	4	13,957	0.0000473	82,025.30	0.0000450	82,016.91	[0 651 142 292]
	1019	4	23,030	0.0001684	173,573.64	0.0001335	173,230.40	[0 843 884 822]
	2129	4	52,668	0.0000101	755,801.98	0.0000101	755,794.91	[0 1770 1491 364]
	3001	4	138,083	0.0000084	1,501,679.83	0.0000064	1,501,500.25	[1720 1720 1720 1720]
$10^{4}$	11	5	55	0.1215523	28.10	0.1167780	24.90	[7 7 7 7 7]
	13	5	55	0.0943402	35.42	0.0932125	34.07	[12 12 12 12 12]
	15	5	71	0.1077167	54.32	0.0992076	45.31	[0 2 2 14 11]
	17	5	75	0.0738689	70.81	0.0663596	57.15	[0 7 7 14 1]
	19	5	99	0.0605900	74.91	0.0586832	71.08	[18 18 18 18 18]
	21	5	110	0.0569909	94.57	0.0533827	86.37	[0 2 1 16 4]
	23	5	134	0.0556996	114.34	0.0513021	103.10	[17 17 17 17 17]
	25	5	125	0.0319601	126.34	0.0306470	121.08	[20 20 20 20 20]
	27	5	206	0.0506670	173.66	0.0426908	141.87	[0 20 0 2 20]
	29	5	254	0.0441466	177.54	0.0403895	162.70	[0 6 7 15 25]
50,000	31	5	310	0.0554706	239.45	0.0445367	185.67	[0 25 15 3 9]
$10^{5}$	1069	5	41,010	0.0002368	213,516.31	0.0001766	213,140.01	[557]
	1543	5	120,197	0.0000706	443,983.37	0.0000671	443,932.86	[1389]
50000	1543	5	120,197	0.0000706	443,983.37	0.0000671	443,932.86	[1389]
	2129	5	224,146	0.0000895	845,357.90	0.0000754	845,006.63	[1173 1173 1173 1173 1173]
	3001	5	276,749	0.0000236	1,678,784.60	0.0000224	1,678,729.13	[1558 1558 1558 1558 1558]
	4001	5	386,491	0.0000099	2,983,681.30	0.0000096	2,983,660.35	[2533 2533 2533 2533 2533]
$10^{6}$	2129	5	224,146	0.0000895	845,357.90	0.0000754	845,006.63	[1173 1173 1173 1173 1173]
	3001	5	276,749	0.0000236	1,678,784.60	0.0000226	1,678,728.62	[0 95 863 1139 720]
	4001	5	386,491	0.0000099	2,983,681.30	0.0000096	2,983,660.35	[2533 2533 2533 2533 2533]
$10^{5}$	5003	5	344,049	0.0000141	4,665,155.64	0.0000132	4,665,001.28	[0 4446 4815 4068 4393]
	6007	5	814,440	0.0000063	6,725,006.06	0.0000062	6,724,984.97	[5451 5451 5451 5451 5451]
	8191	5	1,823,567	0.0000046	12,503,683.85	0.0000040	12,503,472.17	[6214 6214 6214 6214 6214]
$10^{5}$	3997	7	1,434,002	0.0009210	3,528,607.04	0.0007423	3,523,303.69	[0 1715 672 440 232 3237 1928]
50,000	3997	7				0.0007460	3,523,306.21	[0 2367 478 1948 819 3938 3095]
	11,215	7	5,820,266	0.0001360	27,738,414.42	0.0001154	27,733,553.77	[0 7173 10558 4080 3009 5621 2387]
	15,019	7	13,364,583	0.0000214	49,737,519.29	0.0000202	49,736,916.77	[0 8596 7317 11883 2266 12803 7990]
50,000	3997	8	1,951,944	0.0015733	3,771,719.97	0.0012800	3,766,576.88	[0 2896 272 2747 2776 2399 3598 516]
$10^{5}$	3997	8				0.0012820	3,766,577.57	[0 2888 3448 685 555 2157 560 3164]
$10^{5}$	4661	10	3,565,998	0.0068455	5,729,496.43	0.0053282	5,726,228.42	[0 4647 3322 1819 3264 2552 3289 4447 3926 2730]
$10^{6}$	4661	11	3,568,302	0.0129569	6,009,019.34	0.0109750	6,005,710.12	[0 3453 274 174 2376 1064 915 3375 3619 1790 1527]
50,000	4661	11				0.0104903	6,005,723.50	[0 1585 1490 4083 120 2797 754 3846 1690 759 759]
	13,587	11	34,729,142	0.0045143	51,038,555.63	0.0042321	51,026,473.39	[0 6556 7402 4150 12,363 3013 1700 8317 10,104 6757 10,277]
	24,076	11	91,049,568	0.0036279	160,274,172.88	0.0031430	160,214,837.54	[0 2924 9970 14,063 10,282 1365 17,773 7813 22,065 15,267 23,322]
30,000	24,076	11				0.0031374	160,214,923.47	[16,454 16,454 16,454 16,454 16,454 16,454 16,454 16,454 16,454 16,454 16,454]

$d_{2}\left(\cdot \right)$ of GGLP is omitted since it is unchanged in all cases. The blank cells are the same as the above ones.
